# Role of the Rhamnosyl Residue of Ouabain in the Activation of the Na,K-ATPase Signaling Function

**DOI:** 10.3390/life13071500

**Published:** 2023-07-02

**Authors:** Ilya V. Rogachevskii, Dmitriy M. Samosvat, Valentina A. Penniyaynen, Vera B. Plakhova, Svetlana A. Podzorova, Ke Ma, Georgy G. Zegrya, Boris V. Krylov

**Affiliations:** 1 Pavlov Institute of Physiology of the Russian Academy of Sciences, 199034 Saint Petersburg, Russia; 2 Ioffe Institute of the Russian Academy of Sciences, 194021 Saint Petersburg, Russia; 3 Department of Pain Management, Xinhua Hospital, Shanghai Jiaotong University School of Medicine, Shanghai 200240, China

**Keywords:** ouabagenin, ouabain, rhamnosyl residue, Na,K-ATPase, Na_V_1.8 channel, patch-clamp method, organotypic tissue culture method, docking, nociception, analgesics

## Abstract

**Simple Summary:**

Medicinal treatment of chronic pain prompts finding novel approaches to the creation of safe and effective analgesics. Ouabain, a cardiotonic steroid detected in the human organism at extremely low concentrations, has been previously shown by us to switch off the pain signal produced by peripheral neurons. The current manuscript elucidates the mechanism of ouabain binding to its molecular target, the Na,K-ATPase enzyme. Application of very sensitive physiological techniques demonstrated a complete loss of the ouabain effect upon removal of the rhamnosyl residue, a structural element of the ouabain molecule. Theoretical modeling made it possible to determine the contribution of the rhamnosyl residue to the process of ouabain binding with Na,K-ATPase. It was concluded that intermolecular bonds between the rhamnosyl residue of ouabain and Na,K-ATPase amino acid residues identified through modeling are required for the analgesic effect of ouabain to manifest itself. To facilitate creation of fundamentally new safe and effective analgesics, the mechanisms of their binding to the corresponding molecular receptors should be clarified at the atomic level.

**Abstract:**

The signaling or non-pumping Na,K-ATPase function was first observed by us in the nociceptive neuron; Na,K-ATPase transduced the signals from the opioid-like receptors to Na_V_1.8 channels. This study elucidates the role of the rhamnosyl residue of ouabain in the activation of the Na,K-ATPase signaling function. The effects resulting from activation of Na,K-ATPase signaling by the Ca^2+^ chelate complex of ouabain (EO) are not manifested upon removal of the rhamnosyl residue, as demonstrated in viable cells by the highly sensitive patch-clamp and organotypic cell culture methods. Docking calculations show that the rhamnosyl residue is involved in five intermolecular hydrogen bonds with the Na,K-ATPase α1-subunit, which are fundamentally important for activation of the Na,K-ATPase signaling function upon EO binding. The main contribution to the energy of EO binding is provided by its steroid core, which forms a number of hydrogen bonds and hydrophobic interactions with Na,K-ATPase that stabilize the ligand–receptor complex. Another critically important role in EO binding is expected to be played by the chelated Ca^2+^ cation, which should switch on strong intermolecular ionic interactions between the EO molecule and two α1-Na,K-ATPase amino acid residues, Glu116 and Glu117.

## 1. Introduction

For almost a quarter of a century, attention was drawn to a novel function of Na,K-ATPase (NKA). This integral membrane enzyme is present in higher eukaryotes, where it is responsible for the classical ion-pumping function discovered by Skou in the late 1950s [1,2]. At the turn of the current century, the first data began to appear indicating that NKA performs another physiologically relevant function in the cells of various tissues. The non-pumping or signaling NKA function, fundamentally different from its pumping function, was first observed in cardiac myocytes [3,4]. In previous studies, we were able to independently detect this function in sensory neurons [5,6]. Instead of G-proteins that are not involved in the process, NKA was shown to transduce the signals from the opioid-like receptors to a certain subtype of tetrodotoxin-resistant sodium channels (now known as Na_V_1.8 channels) in the sensory neuron membrane [5]. Numerous studies performed during the last 20 years indicate that NKA may represent a novel class of cell-surface receptors [7].

No less mysterious was the discovery of endogenous ouabain (EO) in the bovine hypothalamus and mammalian bloodstream at extremely low concentrations of the nanomolar range [8,9,10]. Ouabain, a cardiac glycoside containing one rhamnosyl sugar moiety attached to the steroid core, has long been applied medicinally to treat congestive heart failure and arrhythmia. Exogenous administration of ouabain at micromolar concentrations results in specific partial inhibition of the NKA pumping function, which forms the basis for its cardiotonic effect, but also accounts for its toxicity in the indicated concentration range.

The analgesic effect of ouabain has never been reported. However, the crosstalk between the non-pumping function of NKA and slow sodium Na_V_1.8 channels made us take a fresh look at the possible physiological role of EO. At the concentrations corresponding to those of EO, ouabain has been demonstrated to modulate the functional activity of Na_V_1.8 channels that encode nociceptive information in warm-blooded animals [11]. It is now well known that Na_V_1.8 channels are considered as markers of nociceptive neurons [12]. We suggest that EO might be another marker of these sensory neurons, which is supported by our recent data. The dose dependence of the inhibitory effect of EO on neurite growth, approximated by the Hill equation, gives an extremely low Kd value (0.1 nM) [11]. This value is an order of magnitude smaller than the Kd value obtained in the patch-clamp experiments, where the effective charge (Z_eff_) transferred by the Na_V_1.8 channel activation gating system was measured during EO action [6,11,13].

**Figure 1:**
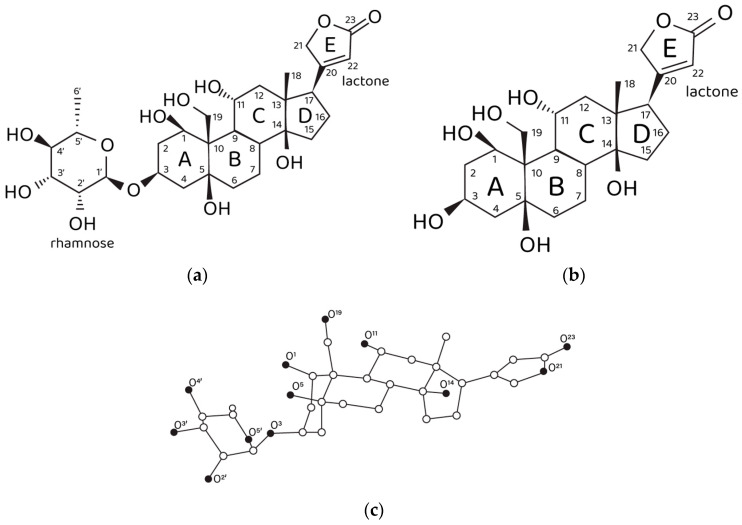
Structural data on ouabain and ouabagenin. (**a**) Chemical structure of ouabain and (**b**) ouabagenin. (**c**) Three-dimensional structure of ouabain and numbering of oxygen atoms. Carbon, white spheres; oxygen, black spheres. Hydrogen atoms are not shown.

On the other hand, ouabain has long been known as the NKA marker. A tempting hypothesis is that the structural features of the ouabain molecule (Figure 1a,c) determine its ability to modulate two different physiological functions of NKA, i.e., the pumping and non-pumping (signaling) functions. Ouabain contains an unusually high number of hydroxyl groups for a cardiotonic steroid (CTS), which opens up a possibility that the endogenous physiologically relevant molecular form of ouabain (EO) is its Ca^2+^ chelate complex. Indeed, it has been demonstrated by the organotypic tissue culture method that the inhibiting effect of ouabain on neurite growth is not manifested in the presence of EGTA, the specific Ca^2+^ chelating agent [11]. Correspondingly, we have suggested that the NKA pumping function is inhibited by exogenous administration of free ouabain in the micromolar range of concentrations, while the NKA signaling function is controlled by subnanomolar and nanomolar concentrations of EO. It should be stressed once again that EO is the Ca^2+^ chelate complex of ouabain, i.e., these two molecules are distinct from each other, though sharing similar geometry. However, the chelation of a Ca^2+^ cation by ouabain introduces an electrophilic moiety into the steroid molecule. In a way, the chelated Ca^2+^ can be considered as a fixed positive point charge that is expected to be electrostatically compensated by the formation of intermolecular ionic bonds upon binding of EO to the NKA molecule. Hence, we suggest that these electrostatic interactions are required to activate the NKA signaling function. Another special role in the process of ligand–receptor EO binding might be played by the rhamnosyl residue since it contains four oxygen atoms that can participate in Ca^2+^ chelation and form intermolecular hydrogen bonds with NKA. Ouabagenin is the aglycone of ouabain, its molecule lacks the rhamnosyl residue and contains a hydrogen atom instead (Figure 1b). The effects of ouabagenin on NKA are investigated herein within the framework of a complex methodological approach that includes calculational methods, the patch-clamp method and the organotypic tissue culture method, which make it possible to elucidate the role of the rhamnosyl residue in modulation of the NKA signaling function by EO. It is necessary to emphasize that the experimental patch-clamp results are obtained on the identified specialized cells, the nociceptive neurons. This suggests a special role of EO in modulation of the CNS signaling, namely, in modulation of its antinociceptive physiological function.

Once again, our suggestion that EO might be the Ca^2+^ chelate complex of ouabain is based on the following results. It has been shown by ab initio calculations that both ouabain and ouabagenin molecules can effectively chelate a Ca^2+^ cation, and two possible chelation modes were detected for each molecule [11,14,15]. Patch-clamp experiments carried out on nociceptive neurons of rats confirmed that nanomolar concentrations of ouabain in an excessive presence of Ca^2+^ modulate the effective charge (Z_eff_) transferred by the Na_V_1.8 channel activation gating system, which was attributed to activation of the NKA signaling function [6,11,13]. Due to the method’s limitations, the direct effects of Ca^2+^ chelation cannot be investigated by the patch-clamp method, because it is technically impossible to obtain reliable results in a Ca^2+^-free environment. The very sensitive organotypic tissue culture method was therefore applied to demonstrate that the inhibitory effect of ouabain on neurite growth, another manifestation of the NKA signaling, is not exhibited upon removal of Ca^2+^ from the culture medium [11].

To address the functional role of the rhamnosyl residue in binding of EO to NKA, it is necessary to compare the effects of ouabain and ouabagenin within a common experimental methodology, the results of which obtained by the patch-clamp and organotypic tissue culture methods are presented herein. Computational approach to the problem requires docking of both molecules with the NKA molecule to provide a detailed insight at the atomic level on the contribution of the rhamnosyl residue to their ligand–receptor binding process. Moreover, docking of the Ca^2+^ chelate complexes of ouabagenin could help elucidate the role of Ca^2+^ chelation in the absence of the rhamnosyl residue. However, a single NKA model would not suffice, because the α1-NKA affinity for ouabain and other CTSs is known to be three orders of magnitude lower in rodents (α1R-NKA, resistant) as compared with other mammals (α1S-NKA, sensitive) [16]. Analyzing the results of ligand docking with both NKA models should make it possible to conclude whether the experimental effects observed on rodent cells result from the resistance of α1R-NKA to CTSs.

Medicinal treatment of chronic pain of various etiologies requires the use of opiates and/or opioids that evoke widely known adverse side effects at the organismal level and are highly addictive. For this reason, the world is experiencing the opioid crisis, which is one of the worst public health crises in history [17,18]. When pain as a sensation becomes chronic, losing its informational and protective function, this pathology is usually corrected only by drug administration. Regretfully, there are no safe and effective analgesics that can replace opiates in the arsenal of clinical medicine.

A very promising approach to help solve this challenging problem is based on the modulation of the functional activity of Na_V_1.8 sodium channels that encode the nociceptive information. It is the high-frequency component of nociceptive neuron impulse firing that transmits information about the pain sensation to the CNS [6]. If this high-frequency component of impulse activity is specifically turned off by a medicinal substance without affecting the signals of other modalities of polymodal nociceptors, such a substance could effectively substitute opiates and opioids in clinical practice.

In our opinion, the opioid crisis could be overcome only by using safe substances of endogenous nature. As an example, a number of short peptides were demonstrated at very low concentrations to modulate the Na_V_1.8 channel activation gating system in nociceptors [19,20]. According to our data, another potentially very effective substance is EO, the Ca^2+^ chelate complex of ouabain, which is also capable of modulating the Na_V_1.8 channels via activation of the NKA signaling function (Kd = 1 nM) [13,21].

On the other hand, the free ouabain molecule (OUA) is known to modulate the NKA pumping function in the micromolar range of concentrations, and there is only one identified CTS binding site within the α1-NKA subunit. CTS binding has been demonstrated to hardly cause any changes in the overall NKA structure, which is supported by the results presented herein, and the binding mechanism is a conformational selection rather than induced fit [22]. Therefore, it is reasonable to expect a substantial difference between EO and OUA in intermolecular interactions formed in their ligand–NKA complexes. At the structural level, it has been shown that Ca^2+^ chelation by OUA and ouabagenin (OBG) does not significantly change the ligand geometry [11,14,15]. However, the introduction of a fixed positive charge in the form of a chelated divalent Ca^2+^ cation drastically affects the ligand electrostatic properties and would require the cation charge to be compensated by ionic interactions with NKA in the ligand–receptor complex. It remains unclear though, whether Ca^2+^ chelation by the ligand is the necessary and sufficient factor that should make the activation of the NKA signaling function possible as a result of ligand binding. The present manuscript demonstrates that the rhamnosyl residue of OUA is another structural factor required to trigger the NKA signaling.

## 2. Materials and Methods

### 2.1. Ligand Docking to NKA

**Figure 2:**
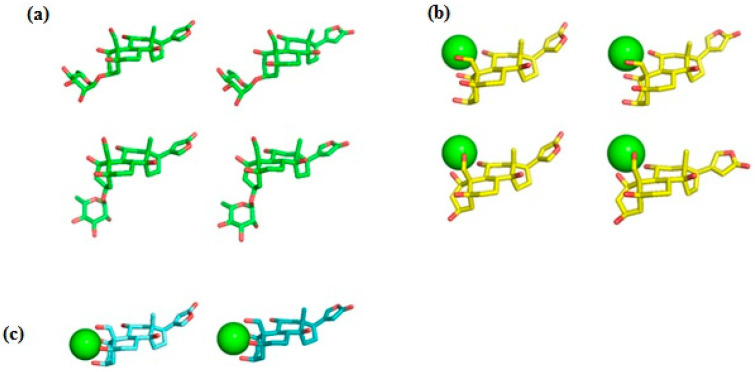
Ligand conformations used for docking with NKA. (**a**) Ouabain (OUA). Carbon, green; oxygen, red. Upper row, the ring A adopts the *chair* conformation; lower row, the *twist* conformation. Ouabagenin (OBG) conformations are essentially the same as those of OUA, but without the rhamnosyl residue. (**b**) Ca^2+^ chelate complex of ouabagenin (OBG-Ca_3_). Carbon, yellow; oxygen, red; calcium, green sphere. Upper row, the ring A adopts the *chair* conformation; lower row, the *twist* conformation. (**c**) Ca^2+^ chelate complex of ouabagenin (OBG-Ca_4_). Carbon, cyan; oxygen, red; calcium, green sphere. The ring A adopts the *chair* conformation. Hydrogen atoms are not shown.

Ab initio RHF/6-31G* conformational analysis of the free ouabain (OUA) and ouabagenin (OBG) molecules, as well as the ouabagenin–Ca^2+^ chelate complex (OBG-Ca), was previously performed [11,14,15]. Four stable conformations were detected for both OUA and OBG, differing by the conformation of the ring A (the *chair* or the *twist*) and orientation of the lactone ring E, which is allowed to rotate freely with respect to the steroid core. For OBG-Ca, six conformations and two possible modes of cation chelation were identified: Ca^2+^ can be chelated either by four (O^1^, O^3^, O^5^, O^19^) or by three (O^1^, O^11^, O^19^) oxygen atoms. The respective chelation modes were designated further as OBG-Ca_4_ and OBG-Ca_3_. In the latter case, the ring A can adopt either the *chair* or the *twist* conformation. The RHF/6-31G* geometry of all 14 conformations was additionally fully optimized at the UHF/cc-pVTZ level of theory using the Orca 5.0 program system [23,24] to obtain molecular structures for docking with NKA. Selected ligand conformations are presented in Figure 2.

The pig ouabain-sensitive α1-NKA isoform (α1S-NKA) in the E2P state characterized by a higher affinity of ouabain binding in comparison with other NKA conformational states was used (PDB code 4HYT) [25]. To obtain the model of the rat ouabain-resistant NKA α1-subunit (α1R-NKA), amino acid residues Gln111 and Asn122 were correspondingly substituted for Arg111 and Asp122 in the initial structure. It is known that resistance of rodent α1-NKA to ouabain is mainly due to the above substitution [16]. The bound ligands present in both models were removed, hydrogens were added, and the entire structures were minimized in UFF forcefield [26] using OpenBabel 2.4. As the ligand position is available from the original structure, we used its geometry center as the center of a cubic 50 Å box. The box size was chosen roughly as the triple maximal linear size of the ouabain molecule, 16 Å. Local docking was carried out within this box 5 times for each ligand conformation using AutoDock Vina [27]. The exhaustiveness was set at 32. Only docking modes with correct lactone ring positioning were included in the further analysis performed with AutoDockTools [28]. The ligand–receptor complexes obtained by docking were once again minimized in UFF forcefield using OpenBabel 2.4.

### 2.2. Patch-Clamp Method

Experiments were conducted on dissociated sensory neurons of newborn Wistar rats applying the short-term cell culture method. Dorsal root ganglia (DRG) isolated from the L5–S1 region of the spinal cord of two newborn rats were processed to obtain the cell culture for each single day of the experiments, as described in detail elsewhere [6,11,21]. The time of enzymatic treatment was dependent on animal age, from 2 to 5 min at 37 °C [29]. The total number of rats utilized to register the data presented herein was 42. The compositions of the extracellular and intracellular solutions used when studying the Na_V_1.8 currents were described earlier [11,19,30]. The whole-cell recording configuration of the patch-clamp method [31] was implemented using the hardware–software setup that comprised the patch-clamp L/M-EPC 7 amplifier, the digital–analog and analog–digital converters.

To study the OBG effects, the agent was added to the extracellular solution at the concentration of 10 nM, which is the same as the concentration of EO earlier demonstrated to modulate the NKA signaling function [11]. Families of Na_V_1.8 currents registered without OBG were taken as control data.

A remarkable sign that it was exclusively the Na_V_1.8 currents that we recorded in the control experiments before the application of the agent under investigation is the fact that the amplitude value of the I_Na_s_ -E function was observed at E ≈ 0 mV, which is the characteristic feature of the Na_V_1.8 channels in the voltage-gated sodium channels superfamily [11,32]. If this criterion was not met, the experiment was interrupted and a new neuron was studied. At the next step, the logarithmic voltage sensitivity functions L(E) before and after the agent application were constructed to evaluate the effective charge (Z_eff_, in elementary charge units, e_0_) transferred by the Na_V_1.8 channel activation gating system and to estimate the changes in Na_V_1.8 channel voltage sensitivity using the Almers theory [33]. The logarithmic limiting conductivity function L(E) is constructed on the basis of a simple expression:

lim L(E) = lim ln(G_Na_s_(E)/(G^max^_Na_s_ − G_Na_s_(E)) → const * exp(Z_eff_ * e_0_ * E/kT),

E→−∞  E→−∞      E→−∞

where G_Na_s_(E) is the voltage dependence of Na_V_1.8 channel chord conductivity, and G^max^_Na_s_ is its maximum value. According to the Almers theory, Z_eff_ can be easily evaluated from the tangent of the slope of the asymptote passing through the very first points of the L(E) function due to the application of Boltzmann distribution. This approach is described in more detail elsewhere [5,6,11,19,21].

The accuracy of Z_eff_ evaluation strongly depends on the correctness of the patch-clamp method application. Both dynamic and stationary errors of the patch-clamp method are determined by the series resistance R_S_, calculated automatically during the experiment. Its value should be less than 3 MΩ because the stationary and kinetic parameters of the currents are otherwise obtained with large errors, as demonstrated by theoretical analysis of limitations of the patch-clamp method applicability [34].

### 2.3. Organotypic Tissue Culture Method

The objects of the study were DRG, cardiac, retina, skin, and liver tissue explants of 10–12-day old White Leghorn chick embryos obtained as described previously [32,35]. Briefly, explants were cultured in a CO_2_ incubator (Sanyo, Osaka, Japan) for 3 days on collagen substrates in Petri dishes at 37 °C and CO_2_. The culture medium consisted of Hank’s solution (45%), EMEM (40%), supplemented with 10% FBS, L-glutamine (2 μM), insulin (0.5 U/mL), glucose (0.6%), and gentamicin (100 U/mL). OBG (10 µM) and EGTA (1 mM) were added to experimental dishes. All chemicals were purchased from Sigma (Sigma-Aldrich, St. Louis, MA, USA). The growth of explants was monitored using a phase contrast microscope starting 24 h after the start of cultivation. An Axio Observer Z1 microscope (Carl Zeiss, Oberkochen, Germany) was used to visualize the explants. Morphometric evaluation of explants was carried out using ImageJ (National Institutes of Health, Bethesda, MD, USA) and ZEN_2012 (Carl Zeiss, Germany) software.

After three days of culturing, two differential zones can be distinctly visualized both in the control and experimental explants: the central zone area (the initial area of explant) and the peripheral growth zone formed due to cell migration and proliferation (visualized as a characteristic halo around the central zone). The area index (AI) was calculated as the ratio of the peripheral growth zone area to the central zone area. The DRG peripheral growth zone is predominantly composed of growing neurites, to a much lesser extent proliferate glia, fibroblasts, and single migrating neurons, whereas the neuron bodies remain in the central zone. In the cardiac tissue, the growth zone contains mostly cardiomyocytes and fibroblasts. In the retinal tissue, the growth zone is formed due to proliferation of ganglion cells, photoreceptor cells, pigment epithelial cells, and some number of fibroblasts. The growth zone of skin explants consists of keratinocytes with varying degrees of differentiation and a small number of fibroblasts. In the liver tissue, the growth zone is comprised mostly of hepatocytes with a small number of fibroblasts. Experiments were conducted using the equipment of the Confocal Microscopy Collective Use Center at the Pavlov Institute of Physiology of the Russian Academy of Sciences.

### 2.4. Statistical Analysis

The data were analyzed with STATISTICA 10.0 (StatSoft, Inc., Tulsa, OK, USA) using the Student’s *t*-test and expressed as the mean value ± SEM. *p* < 0.05 was considered statistically significant.

## 3. Results

### 3.1. Docking with NKA

#### 3.1.1. Structural Features of NKA Models

**Figure 3:**
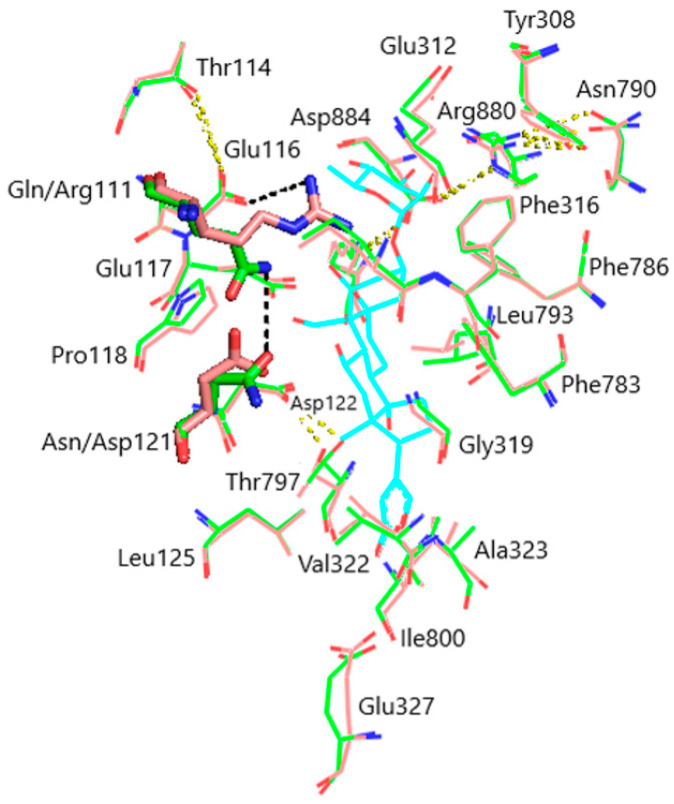
Superimposition of the CTS binding sites in optimized NKA structures. α1R-NKA, green lines (carbon, green; oxygen, red; nitrogen, blue); α1S-NKA, pink lines (carbon, pink; oxygen, red; nitrogen, blue; OUA, cyan lines (carbon, cyan; oxygen, red). Amino acid residues Gln111 and Asn122 in α1S-NKA, and amino acid residues Arg111 and Asp122 in α1R-NKA are displayed as sticks of the corresponding color. Hydrogen bonds are presented with yellow dashed lines. The Gln111–Asn122 bond in α1S-NKA and the Arg111–Glu116 bond in α1R-NKA are presented with black dashed lines. Hydrogen atoms are not shown. RMSD = 0.308.

Models of the CTS binding site in the pig α1S-NKA and rat α1R-NKA subunits are shown in Figure 3. Superimposition of the NKA structures does not demonstrate any substantial shift in the orientation of the amino acid side chains in the CTS binding site, except for that of Gln111/Arg111 and Asn122/Asp122 determining the drastic difference between the non-rodent and rodent α1-NKA affinity to ouabain. Intramolecular interactions between the polar functional groups of NKA amino acid residues participating in CTS binding are presented in Table 1. Two groups were considered to be interacting if the distance between heavy atoms in a possible bond between them did not exceed 4 Å. According to our data, Gln111 and Asn122 are hydrogen bonded in α1S-NKA. In α1R-NKA, Gln111/Arg111 and Asn122/Asp122 substitution favors the formation of Arg111–Glu116 bond, whereas Arg111 and Asp122 are ~5 Å apart, which is too distant to expect a hydrogen bond. Including the direct Arg880–Asp884 bond, four of the observed interactions in both NKA structures involve Arg880 and Asp884 residues, which indicates that their spatial positioning is strongly correlated. The Glu116 and Asp121 carboxylate anions are bound to the Thr114 and Thr797 hydroxyls, respectively. The Glu117, Glu312, and Glu327 carboxylate anions are not demonstrated to interact with other polar residues.

#### 3.1.2. Characteristics of Ligand–Receptor Complexes

Docking of OUA (4 conformations), OBG (4), OBG-Ca_3_ (4), and OBG-Ca_4_ (2) with both NKA models revealed the following. When the ring A in OUA adopts the *twist* conformation, a sterical clash of the rhamnosyl residue with Phe316 prevents the ligand from effective docking to the CTS binding site. Upon docking of OBG and OBG-Ca_3_, changing the ring A conformation does not affect the docking mode. The initial orientation of the lactone ring E with respect to the steroid core was not found to influence the docking position for any of the studied ligands. Moreover, none of the OBG-Ca_3_ conformations was demonstrated to dock to α1R-NKA. Hence, no more than one effective docking mode in the CTS binding site was detected for each ligand in either of the NKA models.

**Figure 4:**
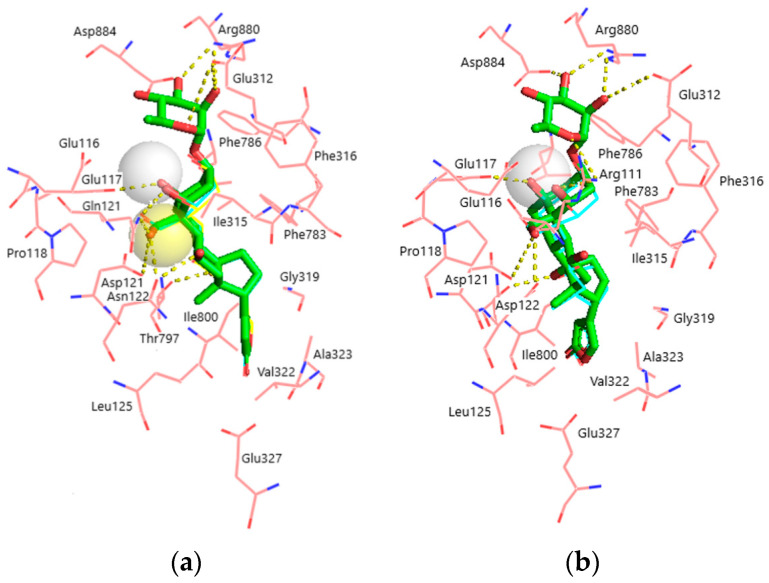
Superimposition of the docked ligands in the CTS binding site. (**a**) α1S-NKA. OUA, green sticks (carbon, green; oxygen, red); OBG-Ca_4_, cyan lines (carbon, cyan; oxygen, red; Ca^2+^, white transparent sphere); OBG-Ca_3_, yellow lines (carbon, cyan; oxygen, red; Ca^2+^, yellow transparent sphere). RMSD = 0.269. (**b**) α1R-NKA. OUA, green sticks (carbon, green; oxygen, red); OBG-Ca_4_, cyan lines (carbon, cyan; oxygen, red; Ca^2+^, white transparent sphere). RMSD = 0.342. NKA is presented with pink lines (carbon, pink; oxygen, red; nitrogen, blue). Ligand–receptor hydrogen bonds are shown with yellow dashed lines. The OBG positions almost totally overlap with those of OUA and are not displayed. Hydrogen atoms are not shown.

Superimposition of the docked ligands in both NKA models is displayed in Figure 4a,b. It is clearly seen that all ligands occupy almost identical positions, except for OBG-Ca_4_ which is shifted ~0.5 Å deeper into the binding site in the α1R-NKA model. Structural parameters of the obtained ligand–receptor complexes are presented in Table 2. OUA is the largest ligand accommodated by 21 NKA residues. OBG lacks the rhamnosyl substituent and contacts 17 NKA residues. Steroid core (rings A-D) oxygen atoms of OUA and OBG form 7 hydrogen bonds with α1S-NKA and only 5 hydrogen bonds with α1R-NKA. Most of these bonds are essentially the same in both models and involve NKA residues located on transmembrane segments M1–M2 of the NKA α-subunit.

#### 3.1.3. Effects of the Rhamnosyl Residue on OUA Binding and Effects of Ca^2+^ Chelation on OBG Binding

According to our results, the rhamnosyl residue of OUA is accommodated by Glu116, Glu312, Arg880, and Asp884 residues in the CTS binding site. The rhamnosyl oxygen atoms form 5 intermolecular hydrogen bonds with Glu312, Arg880, and Asn/Asp122. It is worth noting that Glu116 sterically contacts the rhamnose in both NKA models, but this residue does not participate in intermolecular hydrogen bonds between NKA and the ligands. Its negatively charged side chain carboxylate group, however, participates in an ionic bond with the chelated divalent Ca^2+^ cation in OBG-Ca_4_, which might suggest a special role for Glu116 in binding of OBG-Ca_4_ to NKA. The spatial position of Ca^2+^ in OBG-Ca_4_ favors its electrostatic interaction with the Glu116 carboxylate anion, which cannot occur in OBG. Another negative charge is provided by the Glu117 carboxylate anion. Docking of OBG-Ca_3_ did not reveal any ionic ligand–receptor interactions.

The obtained results correlate with the predicted ligand–receptor binding energy values. A lower affinity of CTSs to α1R-NKA can be accounted for by the fact that the steroid core of the ligands forms 2 hydrogen bonds less with α1R-NKA than it does with α1S-NKA, which makes the ligand–receptor binding ~1.5 kcal/mol less energetically favorable. The rhamnosyl residue forms 5 hydrogen bonds with NKA, and its removal decreases the effect of OBG binding by ~1 kcal/mol in comparison with OUA. The positive charge of Ca^2+^ in OBG-Ca_4_ is compensated by the Glu116 and Glu117 carboxylate anions, which increases the binding energy by ~0.5 kcal/mol. Spatial location of the cation in OBG-Ca_3_ appears to be too distant from the negatively charged NKA functional groups, which totally prevents OBG-Ca_3_ from effective docking with α1R-NKA.

#### 3.1.4. Optimization of Ligand–Receptor Complexes Obtained by Docking

**Figure 5:**
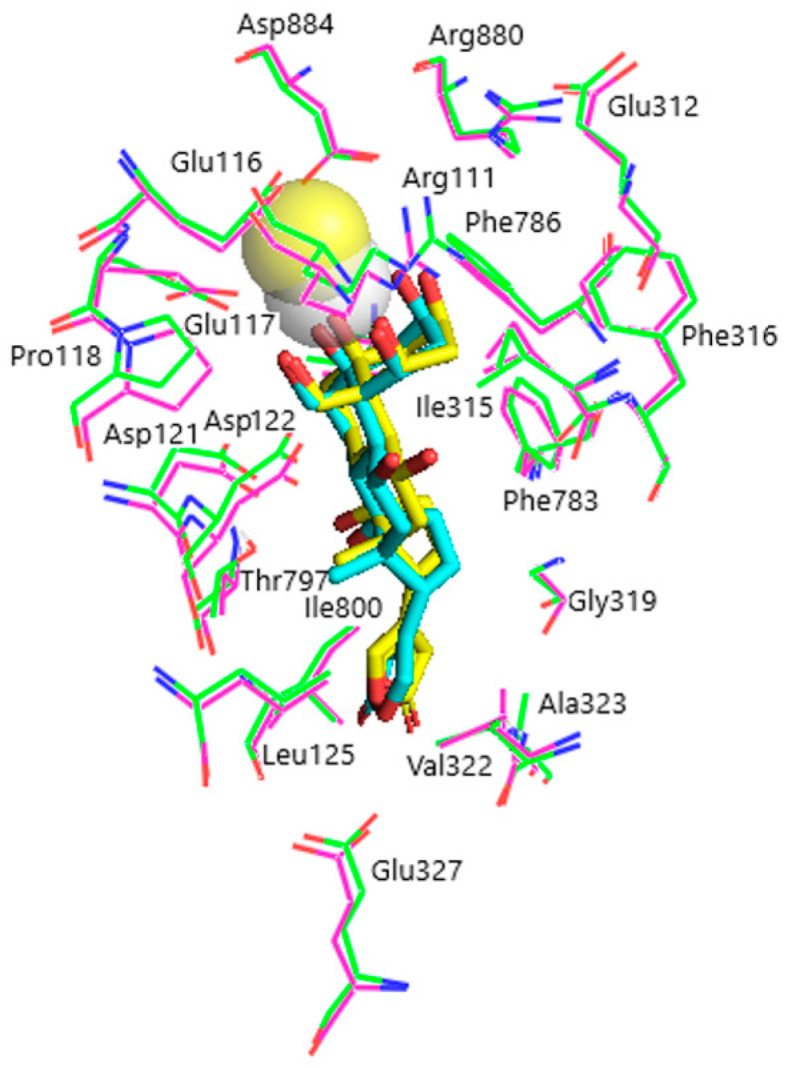
Superimposition of ligand–receptor complexes of OBG-Ca_4_ with α1R-NKA before and after energy optimization. Before optimization, OBG-Ca_4_, yellow sticks (carbon, yellow; oxygen, red); Ca^2+^, yellow transparent sphere; α1R-NKA, green lines (carbon, green; oxygen, red; nitrogen, blue). After optimization, OBG-Ca_4_, cyan sticks (carbon, cyan; oxygen, red); Ca^2+^, white transparent sphere; α1R-NKA, magenta lines (carbon, magenta; oxygen, red; nitrogen, blue). Hydrogen atoms are not shown.

Additional optimization of ligand–receptor complexes obtained by local docking did not result in substantial rearrangement of the ligands in the CTS binding site. The Glu116 carboxylate anion approaches in closer contact with the chelated Ca^2+^, while the Glu117 carboxylate anion moves slightly away from the cation. However, both these functional groups keep compensating the cation positive charge. After optimization, the lactone ring rotates ~30° with respect to the steroid core, and the ligands shift ~0.3–0.5 Å towards the Glu327 carboxylate anion (Figure 5). As a result, a hydrogen bond between the lactone O^23^ oxygen atom and the Glu327 carboxylate oxygen atom could be expected via a water molecule or a proton. Our results on OUA docking with α1S-NKA are in good accordance with the literature [36,37]. The same NKA residues were identified to contact the ligand molecule and the same predicted ligand–receptor binding energy values (−11.0 kcal/mol) were obtained. However, there is a significant disagreement in the α1R-NKA models. In the reported α1R-NKA model Arg111 is bound to Asp122, thus creating a steric hindrance for the ligand to enter the CTS binding site. The Arg111–Glu116 bond observed in our α1R-NKA model does not impose severe steric restrictions on OUA binding, and the ligand docks virtually to the same position as in α1S-NKA. As a consequence, the predicted energy of OUA binding to α1R-NKA is −9.5 kcal/mol, while the value of −7.2 kcal/mol has been published [37]. This may be due to the difference in the protocols of NKA model refinement. We optimized the entire NKA structure, and only local optimization of the CTS binding site has been previously carried out [37].

### 3.2. Patch-Clamp Method

**Figure 6:**
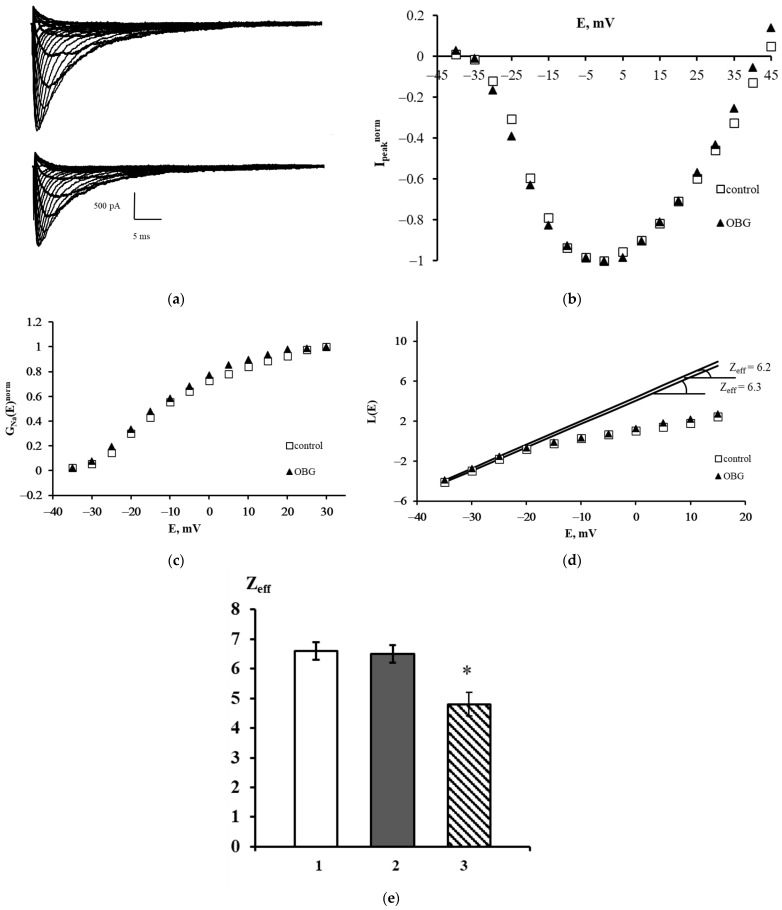
Effect of OBG on the Na_V_1.8 channel. (**a**) Families of Na_V_1.8 currents recorded before (top) and after (bottom) extracellular application of OBG. (**b**) Normalized peak current–voltage functions of the Na_V_1.8 `channel I_peak_^norm^ (E) in the control experiment and after application of OBG. (**c**) Voltage dependence of the Na_V_1.8 channel chord conductance G_Na_^norm^ (E) in the control experiment and after the application of OBG. (**d**) Evaluation of Z_eff_ from the logarithmic voltage sensitivity function L(E) after the application of OBG. The test potential was changed from –60 mV to 45 mV with a step of 5 mV. The holding potential of 300-ms duration was equal to –110 mV in all records. The leakage and capacitive currents were subtracted automatically. (**e**) The effective charge values in control conditions (1) Z_eff_ = 6.6 ± 0.3 (*n* = 18), after the application of OBG (2) Z_eff_ = 6.5 ± 0.3 (*n* = 21) and after the application of EO (3) Z_eff_ = 4.8 ± 0.4 (*n* = 15) [11]. Data are presented as mean ± SEM. A statistically significant difference between the control and experimental values is designated with asterisk (*p* < 0.05).

The families of Na_V_1.8 currents recorded in a control experiment and after extracellular application of OBG are shown in Figure 6a. A slight decrease in the amplitudes of the currents was observed after exposure to OBG, which can be explained by the run-down effect. This effect is an intrinsic feature of sodium voltage-dependent currents recorded in the whole-cell patch-clamp configuration. The peak current-voltage function I_peak_^norm^ (*E*) was obtained using the data presented in Figure 6a. It is clearly seen that OBG does not affect the shape of the left branch of this function and does not shift its amplitude value (Figure 6b). The voltage dependencies of the chord conductance also remain unchanged after the application of OBG (Figure 6c). When the chord conductance dependencies are obtained, Z_eff_ can be evaluated from the *L*(*E*) function constructed in accordance with the Almers limiting slope method [33]. The Z_eff_ values in an exemplary control experiment and after the application of OBG were 6.3 and 6.2 elementary charge units, respectively (Figure 6d). Figure 6e summarizes the patch-clamp data regarding the effects of OBG on the Na_V_1.8 channel. The mean Z_eff_ values in control experiments and after extracellular application of OBG (10 nM) are presented. Obviously, OBG does not statistically significantly modulate the effective charge transferred by the Na_V_1.8 channel activation gating system. Thus, our patch-clamp experiments have demonstrated that OBG applied at the same concentration as EO previously [6,11] does not activate the NKA signaling function. At the tissue level discussed below, the OBG effect was observed only when its concentration was as high as 10 µM which is due to inhibition of the NKA pumping function by the agent.

### 3.3. Organotypic Tissue Culture

**Figure 7:**
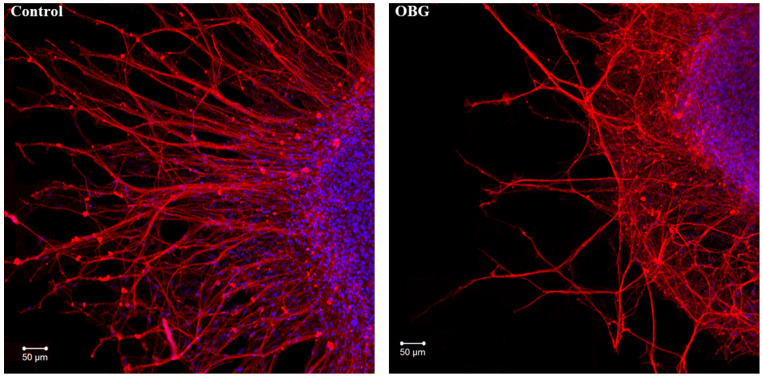
OBG effects on DRG neurite growth. Fragments of the DRG explant growth zone in control conditions (**left**) and after application of OBG at 10 µM (**right**), third day of culturing. Both control and OBG-treated neurons were immunostained with anti-neurofilament 200 antibody (red). Nuclei were counterstained with DAPI (blue). Scale bar = 50 μm.

**Figure 8:**
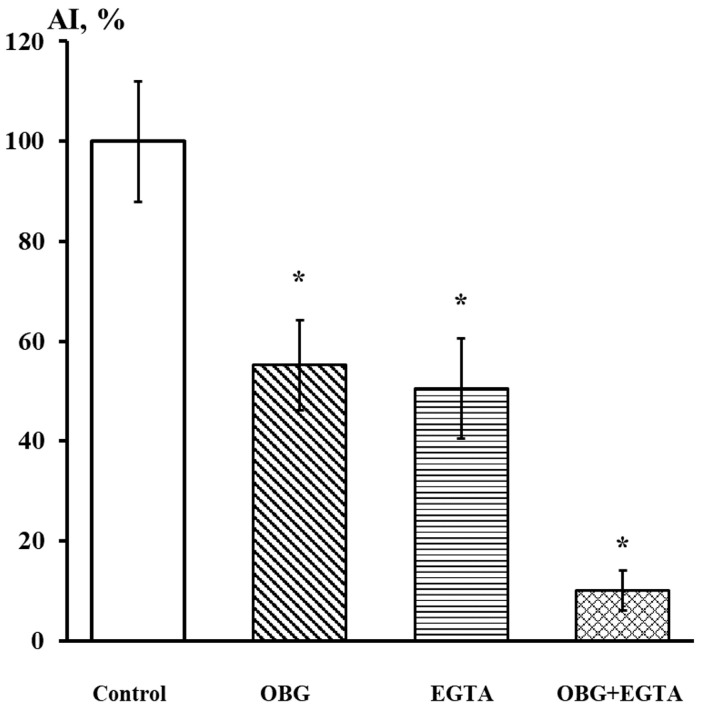
EGTA at 1 mM dramatically enhances the neurite-inhibiting effect of OBG at 10 µM. Ordinate axis, area index (AI, %). Statistically significant differences between the control and experimental values are designated with asterisks (*p* < 0.05).

Fragments of the peripheral growth zone in an exemplary DRG explant in the control conditions and after exposure to OBG (10 µM) are displayed in Figure 7. OBG inhibited the neurite growth by 50 ± 6% (*n* = 24, where *n* is the number of explants, *p* < 0.05), which indicates that the applied concentration is close to the Kd value (Figure 8). It should be stressed that the Kd value for OBG obtained by the organotypic tissue culture method is 5 orders of magnitude higher than that of EO (0.1 nM) [11,13]. Introduction of EGTA (1 mM), the selective calcium chelating agent, to the culturing medium also resulted in inhibition of the neurite growth by 50 ± 6% (*n* = 20, *p* < 0.05) (Figure 8). When DRG were cultured in the media containing both EGTA (1 mM) and OBG (10 µM), the neurite growth was inhibited by 90 ± 7% (*n* = 27, *p* < 0.05) (Figure 8). Virtually total inhibition of DRG neurite growth as a result of combined EGTA and OBG application is fundamentally different from the reported effect of EGTA (1 mM) and EO (0.1 nM) [11]. The neurite-inhibiting effect of OBG is not correlated with the presence in the culturing media of Ca^2+^ required to activate the NKA signaling function, and can be accounted for by inhibition of the NKA pumping function.

**Figure 9:**
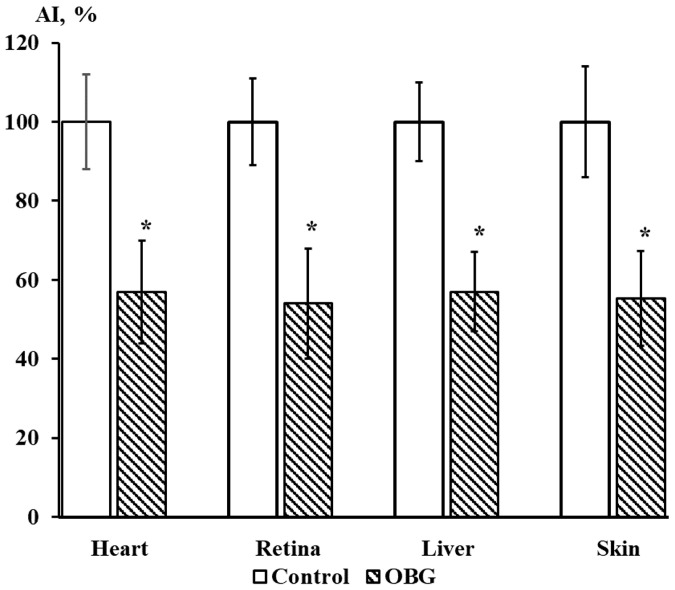
OBG effects (10 µM) on cardiac, retina, liver, and skin tissue explants growth. Ordinate axis, area index (AI, %). Statistically significant differences between the control and experimental values are designated with asterisks (*p* < 0.05).

Cardiac, retina, liver, and skin tissue explants growth were also inhibited by OBG (10 µM) by 43 ± 9% (*n* = 27, *p* < 0.05), 46 ± 8% (*n* = 28, *p* < 0.05), 43 ± 5% (*n* = 26, *p* < 0.05) and 44 ± 7% (*n* = 26, *p* < 0.05), respectively (Figure 9). The data obtained indicate that the OBG effect on growth and proliferation of explants is not tissue-specific.

## 4. Discussion

The present manuscript aims to provide a more detailed insight into the molecular mechanism of EO binding to α1-NKA; more precisely, into the structural factors that determine the unique specificity of this molecular mechanism. It would make sense to nominally divide the EO molecule into three parts: the rhamnosyl residue, the chelated Ca^2+^ cation, and the steroid core, which is exactly the same in EO, OUA, and OBG. OUA and OBG occupy essentially the same position in the CTS binding pocket, and their steroid cores are involved in several hydrogen bonds with NKA, which stabilize the ligand–receptor complexes and, together with hydrophobic interactions of the steroid core with non-polar amino acid residues, provide the main contribution to the binding energy of the ligands to NKA. As demonstrated herein, these bonds are the same for OUA and OBG, though the steroid cores of the ligands form seven hydrogen bonds with α1S-NKA and only five hydrogen bonds with α1R-NKA, which accounts for the α1R-NKA resistance to CTS (Table 2). Consequently, the role of the rhamnosyl residue in activation of the NKA signaling function by EO can be elucidated by comparing the effects of OUA and OBG, while the role of the Ca^2+^ cation can be clarified by analyzing differences between the effects of OBG and its chelates OBG-Ca_4_ and OBG-Ca_3_. A relatively simple calculational protocol applied in the present study can help solve this problem theoretically, but it is very challenging technically to verify the theoretical considerations by experimental methods.

Our experimental patch-clamp data show that OBG does not trigger the NKA signaling function and, therefore, does not modulate the effective charge transfer of the Na_V_1.8 channel activation gating system. The activation gating system charge displacement of sodium channels was for the first time observed on the Na_V_1.1 channels using the complicated method of displacement currents recordings [38]. The main disadvantage of this method is that the cell membrane contains many other proteins, the charged components of which can reduce the accuracy of measuring the effective charge transfer. A fundamentally new approach, free from the above drawback, was elaborated by Almers [33]. Unfortunately, the Almers method has its limitations, which make it impossible to measure the gating charge transfer of many ion channels, including Na_V_1.1, but this method is perfectly applicable to investigate the electrophysiological behavior of Na_V_1.8 channels [6]. A remarkable feature of these channels is that the amplitude value of the Na_V_1.8 channel peak current–voltage function should be observed at E ≈ 0. All other members of the voltage-gated sodium channels superfamily, including tetrodotoxin-resistant channels, do not meet this criterion. In addition, all other tetrodotoxin-resistant sodium channels (Na_V_1.6, Na_V_1.7, Na_V_1.9) differ sharply from the Na_V_1.8 channels in the relaxation behavior of their gating systems [12,39]. This allows us to selectively isolate Na_V_1.8 currents for further processing. When other tetrodotoxin-resistant currents affected the recordings, the experiment was interrupted and processing of the neuron responses was terminated. The measurement of the peak current–voltage function and the construction of the voltage dependence of the chord conductivity on its basis unambiguously indicates that effective charge transfer is associated with the functioning of the Na_V_1.8 channels only, and no other sodium channels are involved in the process. The Almers method exploits the relationship of charge movement and chord conductance voltage dependence, yielding the value of effective charge transferred by the gating device of a single channel [6,33]. This method is based on processing of weak nonlinearities of the activation gating system. It is important to emphasize that the discovery of weak nonlinearities, which we previously used to describe the Na_V_1.1 sodium channel inactivation gating device that do not follow a simple single-barrier model, led to the finding of new molecular mechanisms for important physiological phenomena such as adaptation [40].

The results presented in Figure 6 illustrate the application of the Almers method for evaluation of the effective charge transferred by the Na_V_1.8 channel activation gating system in the intact nociceptive neuron in the physiological conditions maximally resembling those that exist in the living organisms of warm-blooded animals. These conditions are critically important for the statistically significant Z_eff_ evaluation due to the specific physiological role of the Na_V_1.8 channels in the nociceptive neuron. These slow sodium channels serve as the effector unit in the receptor- and transducer-mediated signaling cascades triggered by neighboring membrane and submembrane proteins [21]. In the nociceptive neuron, the Na_V_1.8 channels are coupled to Na,K-ATPase which performs here the functions of both the OBG, OUA and EO receptor and the signal transducer. It has been demonstrated in our prior publications that the activation of the Na,K-ATPase signaling function results not only in a decrease in the Na_V_1.8 channel effective charge but also in a shift of the Na_V_1.8 channel current–voltage characteristics to the right along the voltage axis, towards more positive voltage values [32,41]. Such a shift has also been observed as a result of direct interaction of the attacking molecule with the Na_V_1.8 channel activation gating system [20]. Moreover, a non-stationary shift of the current-voltage function due to dynamic perfusion of the intracellular solution [31] necessarily occurs in the course of a patch-clamp experiment conducted according to the methodology applied herein. Partial contributions of these three processes to the value of the current–voltage function shift cannot be quantitatively identified in the experiments, but the physical background of the observed phenomenon can be qualitatively described in the framework of the Gouy–Chapman–Stern theory. According to this theory, the sodium gating mechanism is sensitive to the local transmembrane potential, which is different from the bulk-to-bulk membrane potential due to the surface charges at the external membrane [42,43]. All the abovementioned processes modulate the local electric field in an individual manner for every single experimental cell, evoking an individual shift of the current–voltage function, which makes the averaging of the initial sections of the Almers logarithmic limiting conductivity L(E) function impossible. However, the effective charge value is, of course, independent of the current–voltage function shift, which allows to obtain the Z_eff_ value averaged over a number of experiments. Thus, Figure 6 illustrates all steps of the Almers method application to register and process the Na_V_1.8 channels responses. The distinct feature of this method is not only its ultimate sensitivity, but also its applicability to the investigation of intact viable cells, where all native protein–protein interactions of the Na_V_1.8 channels with their neighboring membrane and submembrane proteins are retained, and the cellular downstream signaling cascades are fully functional and can be activated. The methodology used in the current manuscript is the only one published so far that made it possible to register the effects of OBG, OUA, and EO in the nociceptive neuron.

As opposed to EO applied at the same concentration, OBG (10 nM) does not exhibit any statistically significant effect on Z_eff_ in our patch-clamp experiments, which indicates the importance of the rhamnosyl residue for the target physiological effect of EO: modulation of the effective charge transferred by the Na_V_1.8 channel activation gating system. Because the gigaseal formation does not occur in a Ca^2+^-free extracellular solution, the patch-clamp method cannot provide any direct data regarding the contribution of Ca^2+^ chelation to activation of the NKA signaling function. This difficulty can be overcome using the organotypic tissue culture method, which also allows to examine viable nociceptive neurons and cells of other tissues with very high sensitivity in the presence of Ca^2+^ and in a Ca^2+^-free environment. Both OBG and EO inhibit neurite growth of the nociceptive neurons, and the OBG effect (Kd = 10 µM) is much weaker than that of EO (Kd = 0.1 nM). The Kd values differ by five orders of magnitude, which also supports the idea that the rhamnosyl residue is required to trigger NKA signaling. Elimination of Ca^2+^ from the extracellular solution by EGTA strongly enhanced the neurite-inhibiting effect of OBG applied at the Kd concentration; in fact, almost total suppression of neurite growth has been observed. This effect is very different from that of EO also applied at its Kd concentration; in the latter case, the combined application of EO and EGTA did not enhance the EO effect at all [11,13]. Since EGTA itself is a potent inhibitor of neurite growth, the observed effect should be attributed to the action of EGTA, while the EO effect is apparently not manifested in Ca^2+^-free media. The suggested interpretation of the experimental data indicates the importance of Ca^2+^ chelation for activation of the NKA signaling function, which mediates the inhibition of neurite growth.

According to the docking results, the rhamnosyl residue is in direct contact with four NKA residues, all of which are notably charged (Glu116, Glu312, Arg880, Asp884), and forms five hydrogen bonds with Glu312, Arg880, and Asp884 in both NKA models (Table 2). These hydrogen bonds determine the orientation of the Glu312, Arg880, Asp884 side chains in the ligand–receptor complex of OUA with NKA, which is in general agreement with the available data [44]. Importantly, Glu116 in our models is too distant from the rhamnose oxygen atoms to be involved in polar interactions with the ligand. Conformational freedom of the Glu116 negatively charged side chain carboxylate group and its spatial position is restricted by a hydrogen bond with the Thr114 hydroxyl (Table 1). However, the Glu116 carboxylate anion (together with the Glu117 carboxylate anion) is demonstrated to provide its negative charge to electrostatically compensate the double positive charge of chelated Ca^2+^ upon docking of OBG-Ca_4_. It should be noted that the Ca^2+^ charge remains uncompensated upon docking of OBG-Ca_3_, although Asp121 in both NKA models and Asp122 in α1R-NKA could have adjusted their side chains to accommodate the cation. Hence, a significant penalty on the energy of OBG-Ca_3_ binding to NKA is imposed; the ligand is unable to effectively dock with the α1R-NKA model. These findings could not be expected a priori and convincingly support our suggestion regarding the critical role of Ca^2+^ in EO binding to NKA, which results in activation of the NKA signaling function. The energy of the steroid core (in fact, OBG) binding provides the principal contribution to the total EO binding energy to NKA. Chelation of the Ca^2+^ cation is energetically favorable, if its double positive charge is compensated upon docking due to intermolecular ionic interactions, and additionally stabilizes the ligand–receptor complex. However, it does not seem to be a sufficient factor for activation of the NKA signaling function.

The main and somewhat unexpected result of the present study is a complete inability of OBG to activate the NKA signaling function. Our experimental data obtained on viable cells indicate that the rhamnosyl residue of EO is fundamentally important for the implementation of the mechanism of ligand–receptor binding of this molecule to NKA. Several intermolecular hydrogen bonds with NKA formed by the rhamnosyl residue are responsible for a significant energy contribution, necessarily required to trigger NKA signaling. The role of Ca^2+^ chelation in the mechanism of activation of the NKA signaling function by EO will be discussed in a future manuscript.

## Figures and Tables

**Table 1:** Intramolecular interactions between the polar functional groups of NKA amino acid residues participating in CTS binding and distances between heavy atoms involved in the interactions.

α1S-NKA	α1R-NKA
Gln111–Asn122 (3.8 Å)	Arg111–Gln116 (3.7 Å)
Thr114–Glu116 (3.5 Å)	Thr114–Glu116 (3.4 Å)
Asp121–Thr797 (3.4 Å)	Asp121–Thr797 (2.9 Å)
Tyr308–Arg880 (3.7 Å)	Tyr308–Arg880 (3.1 Å)
Asn790–Arg880 (3.3 Å)	Asn790–Arg880 (3.2 Å)
Arg880–Asp884 (3.5 Å)	Arg880–Asp884 (3.3 Å)
Asp884–Lys905 (2.6 Å)	Asp884–Lys905 (3.1 Å)

**Table 2:** Characteristics of ligand–NKA complexes obtained by docking.

NKA Isoform	α1S-NKA				α1R-NKA		
Ligand	OUA	OBG	OBG-Ca_4_	OBG-Ca_3_	OUA	OBG	OBG-Ca_4_
Interacting NKA residues	Gln111	Gln111	Gln111	Gln111	Arg111	Arg111	Arg111
	Glu116		Glu116		Glu116		Glu116
	Glu117	Glu117	Glu117	Glu117	Glu117	Glu117	Glu117
	Pro118	Pro118	Pro118	Pro118	Pro118	Pro118	Pro118
	Asp121	Asp121	Asp121	Asp121	Asp121	Asp121	Asp121
	Asn122	Asn122	Asn122	Asn122	Asp122	Asp122	Asp122
	Leu125	Leu125	Leu125	Leu125	Leu125	Leu125	Leu125
	Glu312				Glu312		
	Ile315	Ile315	Ile315	Ile315	Ile315	Ile315	Ile315
	Phe316	Phe316	Phe316	Phe316	Phe316	Phe316	Phe316
	Gly319	Gly319	Gly319	Gly319	Gly319	Gly319	Gly319
	Val322	Val322	Val322	Val322	Val322	Val322	Val322
	Ala323	Ala323	Ala323	Ala323	Ala323	Ala323	Ala323
	Glu327	Glu327	Glu327	Glu327	Glu327	Glu327	Glu327
	Phe783	Phe783	Phe783	Phe783	Phe783	Phe783	Phe783
	Phe786	Phe786	Phe786	Phe786	Phe786	Phe786	Phe786
	Leu793	Leu793	Leu793	Leu793	Leu793	Leu793	Leu793
	Thr797	Thr797	Thr797	Thr797	Thr797	Thr797	Thr797
	Ile800	Ile800	Ile800	Ile800	Ile800	Ile800	Ile800
	Arg880				Arg880		
	Asp884				Asp884		
Predicted binding energy to NKA, kcal/mol	−11.0	−10.2	−10.9	−9.7	−9.5	−8.6	−9.1

## Data Availability

Not applicable.

## Abbreviations

EO: endogenous ouabain, the Ca^2+^ chelate complex of ouabain; NKA: Na,K-ATPase; CTS: cardiotonic steroid; Na_V_1.8: slow sodium channel; Kd: the Hill equation dissociation constant; Z_eff_: the effective charge of the Nav1.8 activation gating system; CNS: the central nervous system; α1-NKA: Na,K-ATPase α1-isoform; α1R-NKA, ouabain-resistant Na,K-ATPase α1-isoform in rodents; α1S-NKA, ouabain-sensitive Na,K-ATPase α1-isoform; OUA: ouabain; OBG: ouabagenin, the aglycone of ouabain; OBG-Ca: the Ca^2+^ chelate complex of ouabagenin; OBG-Ca_4_: the Ca^2+^ chelate complex of ouabagenin where the cation is chelated by four oxygen atoms of ouabagenin (O^1^, O^3^, O^5^, O^19^); OBG-Ca_3_: the Ca^2+^ chelate complex of ouabagenin where the cation is chelated by three oxygen atoms of ouabagenin (O^1^, O^11^, O^19^); DRG: dorsal root ganglia; EMEM: Eagle’s minimal essential medium; FBS: fetal bovine serum; E: the value of transmembrane voltage potential; L(E): the logarithmic limiting conductivity function; I_Na_s_-E: the current–voltage function of the Nav1.8 channel; G_Na_s_(E): the voltage dependence of the Na_V_1.8 channel chord conductivity; EGTA: ethylene glycol-bis(β-aminoethyl ether)-*N*,*N*,*N*′,*N*′-tetraacetic acid; AI: the area index.
